# Use of a Smartphone Application Can Improve Assessment of High-Fat Food Consumption in Overweight Individuals

**DOI:** 10.3390/nu10111692

**Published:** 2018-11-06

**Authors:** Agata Chmurzynska, Monika A. Mlodzik-Czyzewska, Anna M. Malinowska, Jolanta Czarnocinska, Douglas J. Wiebe

**Affiliations:** 1 Institute of Human Nutrition and Dietetics, Poznań University of Life Sciences, 60-624 Poznań, Poland; monika.mlodzik@up.poznan.pl (M.A.M.-C.); anna.malinowska@up.poznan.pl (A.M.M.); jolanta.czarnocinska@up.poznan.pl (J.C.); 2 Department of Biostatistics, Epidemiology and Informatics, Perelman School of Medicine, University of Pennsylvania, Philadelphia, PA 19104-6021, USA; dwiebe@upenn.edu

**Keywords:** dietary assessment, food frequency questionnaire, ecological momentary assessment, high-fat products

## Abstract

Background: We evaluated the feasibility of an application for measuring the frequency of consumption of high-fat foods and compared this application with standard methods. Methods: Twenty-six females and thirty six males aged 20–40 were enrolled in Poland. Participants completed the Block Screening Questionnaire for Fat Intake (BSQF; Q1) and a second questionnaire (Q2) with additional high-fat foods. The participants were then monitored for ten days in a real-time manner using a smartphone application that employed the same lists of food as Q2. Results: Most subjects (84%) gave replies to at least three prompts on at least 5 days. The results from Q1 and the application were correlated (r = 0.42, *p* < 0.001). Energy intake and the frequency of consumption of high-fat foods were correlated in the overweight/obese group (r = 0.83, *p* < 0.001). The mean differences between Q2 and the app were similar in both groups but the agreement limits were wider in the overweight/obese group than in the normal weight group. Conclusions: An application for mobile devices is a feasible tool for capturing the frequency of high-fat food consumption and it seems to improve the measured variable, especially in overweight or obese people.

## 1. Introduction

Diet is a major environmental factor that contributes to non-communicable diseases, including cancer, cardiovascular disease, obesity and type-2 diabetes [1,2]. Nutrient intake assessment is essential for national food policies, in the monitoring of individuals’ nutritional status and also in research into associations between diet and health. However, accurate food intake assessment remains a significant challenge in nutritional science [3,4].

The most commonly used methods for food intake assessment are dietary records, 24-h dietary recall and food frequency questionnaires (FFQs). The food records approach provides an overall estimation of food intake but is a very time-consuming method and therefore of limited use, especially in large cohort studies. A major limitation of the 24-h dietary recall method is the day-to-day variability in food intake; this can be partly overcome if the interview is performed more than once. The FFQs are easier and cheaper to administer and process. FFQs contain a defined list of food and beverages and ask about the usual frequency of consumption over the time period queried. In some cases, information on portion sizes is also collected but little information is collected on the additional characteristics of food eaten [5]. Despite these methodological limitations, FFQs have been widely used since the beginning of large-scale nutritional epidemiology studies [6]. Simplified or targeted FFQs—known as brief dietary assessment tools—have also been developed. They are used when an investigator is interested in a single nutrient or a specific food group [5]. Several screeners for fat intake assessment have been designed and validated [7,8,9].

One common error in estimating food intake is underreporting and people may report as little as 60–90% of their actual intake. Moreover, in case of different foods and beverages (e.g., alcohol or snacks), so-called selective underreporting has been observed [10,11]. All these limitations of the traditional methods of food intake assessment warrant the development of new methods that can better reflect real situations.

Technological progress has the potential to enhance dietary assessment methods. Self-reported tools using computer, internet, telecommunications and imaging technologies have been developed [6]. Smartphone applications are one such type of innovative method. Some of these applications are based on Ecological Momentary Assessment (EMA), which enables researchers to collect data on participants’ states in real-time. EMA involves repeated measurements of participants’ experiences and behaviors within their natural environment as they experience it [12]. EMA is thought to be less prone to retrospective self-report bias because participants answer questions about what is happening in the current moment [13]. Despite the promise of these technological advances and despite the strong interest in ecological models of health behavior, there have been very few studies describing and validating EMA apps. Such apps have previously been used to monitor feeding and lifestyle behavior [14,15,16,17] to count eating and overeating events [18] and to identify cues associated with the consumption of sweetened beverages and snacks [19]. Smartphone applications have mostly been used in intervention studies, to render the dietary interventions more effective [20]. Mobile phone dietary assessment methods have shown similar validity and reliability as conventional methods. However, participants’ satisfaction and preferences were higher for mobile phone methods than for conventional methods [21].

Although there have been many studies on how intake of macronutrient affects functioning of the body, much controversy remains about the possible link between fat intake and health [22,23]. This controversy partly results from the overall complexity of nutritional studies *per se* but there are also specific issues that should be considered in studies on understanding the role of fat in pathophysiology of different diseases—in particular, the intake of different types of fats may have different metabolic consequences; food we eat is a complex matrix and nutrients can interact with each other; finally, dietary manipulations always involve multiple variables [22]. The relationships between fat intake and disease thus remain not well understood and this understanding is further hindered by the lack of an appropriate biomarker for total fat intake [24]. For these reasons, improvements in the methods of measuring fat intake could bring significant advancements to nutritional science. To date, no EMA application for fat intake assessment has been designed and tested.

Given all these considerations, hypothesized EMA would be well received by research participants as a method of measuring dietary behavior. This would yield data which would be more detailed and thus more accurate than data collected using traditional measures. The aim of this study was thus to design and evaluate the feasibility of, an application for measuring the frequency of consumption of high-fat foods and to compare this application with standard retrospective methods. Since body weight is one of the factors that may contribute to underreporting of food intake [25], we also considered whether body weight status may differentiate reporting on high-fat food intake and responses to app prompts.

## 2. Materials and Methods

### 2.1. Study Design

Subjects were enrolled in Poznań, Poland for a bigger study focused on associations between fat sensitivity and fat intake. All consecutive participants enrolled between September and October 2016 were included in the application validation study. The subjects were female and male adults between 20 and 40 years of age. The research protocol was approved by the Local Ethics Committee (966/15). All participants gave their written informed consent.

Recruitment was conducted using online advertisements circulated through social media. Secondarily, participants were encouraged to mention the study to friends and family members and to ask them to consider participating, which served as a snowball sampling technique. Exclusion criteria included chronic diseases (e.g., diabetes, metabolic syndrome, cancer, hyporthyroidism), recent dieting or being on a calorie restricted diet, use of medications known to affect taste, body weight, lipid profile and appetite, moderate and heavy smoking (more than one pack per week), shift work and being pregnant or lactating. Eligible participants came in person to the Institute of Human Nutrition and Dietetics at Poznań University of Life Sciences, where all the procedures were conducted.

### 2.2. Anthropometric Measurements

Basic anthropometric measurements were performed. Weight (to 0.1 kg precision) and height (to 0.01 m precision) were measured with a stadiometer and an electronic scale, respectively. Body mass index (BMI) was calculated as body weight in kg divided by height in m squared.

### 2.3. Measurement of the Frequency of Consumption of High-Fat Foods and Food Intake Assessment

Each participant first completed a number of paper questionnaires regarding demographics and eating habits. The initial questionnaire was the Block Screening Questionnaire for Fat Intake (BSQF), which we will refer to as Q1. The BSQF was originally developed by Block [7] to measure high-fat food intake; it consists of a list of 13 products or groups of products: hamburgers/cheeseburgers/meat loaf, beef steaks/roasts, pork, hot dogs, ham/lunch meats, salad dressings/ mayonnaise, margarine/butter, eggs, cheese, whole milk, French fries/fried potatoes, doughnuts/pastries/cake/cookies and white bread/rolls/bagels (including sandwiches). After approximately 1.5 h, subjects were asked to complete the BSQF for a second time in a version with some groups of products listed separately and containing additional high-fat foods. This questionnaire (Q2) contained the following commonly eaten products and groups of products: oil, margarine, lard, butter, mayonnaise/salad sauce, pork, duck/goose meat, fried chicken, fried fish, smoked salmon/eel/mackerel/halibut, bacon, salami, kabanos sausage, regular sausage, pâté, egg yolk, cheese, processed cheese, French fries, pizza, hamburger, cheeseburger, hot dog, baked or fried sandwiches, kebab, pancakes/crepes, potato pancakes, potato chips, doughnuts, pastries, cookies, chocolate, whipped cream, cream (over 15% fat), whole milk, avocado, nuts and peanut butter. The products were selected from a database of Polish food products and based on their fat content [g/g of product] of at least 10%. The measure we derived from these two questionnaires was the total sum of fatty food servings eaten in a period of one week.

Furthermore, participants were monitored prospectively for approximately one week to report the high-fat foods they consumed each day. This was accomplished by providing each participant with a smartphone that contained an app designed for this study. The application was developed by IT Generator. The phone was programmed to prompt the participant at 9 a.m., 1 p.m., 5 p.m. and 9 p.m., with each prompt asking the participant whether they had eaten any food since the previous prompt. If the subject replied Yes, the subject was presented with the list of high-fat foods which was identical as in the Q2 and instructed to choose all the food items that applied. If the subject did not reply, he or she received two reminders 15 and 30 min later; after this time, the application was blocked until the next prompt in the schedule. The participants were monitored starting on a Mondays and asked to begin using the app and responding to prompts on the following day, continuing for at least 7 days in total. After this time, the participants returned for a second visit to the Institute of Human Nutrition and Dietetics. Participants were not able to initiate self-reports. Additionally, there was no feedback from the application, so the participants had no insight in the results.

Finally, the food intake was analyzed using an estimated food record, which was filled in for three days during the follow-up period. The energy content and nutritional value of the daily food rations, as well as the percent of energy from fat, were calculated based on food composition tables using the computer software package Diet 5.0 (Food and Nutrition Institute, Warsaw, Poland).

The participants attended a learning session provided by a qualified dietician, where they were instructed in using the food diary and estimating portion sizes. They were also trained in the use of the application.

### 2.4. Statistical Analysis

Comparisons between the two instruments were made using Pearson’s correlation coefficient. Differences between the BMI subgroups were examined using Student’s *t*-test. A Bland–Altman plot was used to determine the limits of agreement between the two variables by calculating the standard deviation of the difference between the two measures. All statistical analysis was performed using Statistica software and *p* values < 0.05 were considered statistically significant.

## 3. Results

The characteristics of the study participants are presented in Table 1. The median frequencies of consumption of high-fat foods in participants overall were 18.5 for Q1 and 30.5 for Q2. The mean frequencies of consuming high-fat foods, as measured with Q1 and Q2, did not differ significantly between normal weight and overweight/obese subjects.

The high-fat food consumption measured by Q1 corresponded highly with that measured by Q2 (r = 0.68, *p* < 0.001) but the correlation between Q1 and Q2 was higher in the normal weight subjects (r = 0.75, *p* < 0.001) than in the overweight and obese subjects (r = 0.55, *p* < 0.001).

Subjects were compliant with the protocol in terms of being monitored prospectively and reporting to the app. There was no difference in the response rate between normal weight and overweight/obese individuals. The median number of days of follow-up was nine (ranging from four to ten days) and most subjects (52 people, 84% of the group) gave replies to at least three prompts on at least five days. Those data are further referred to here as ‘valid responses’. Data from the entire data set and the subset of valid responses were correlated (r = 0.90, *p* < 0.001). As a lack of response might be the equivalent of forgetting or lack of answers—a limitation of retrospective methods—we analyzed the entire dataset and the subset of valid responses. The valid responses may be more informative in estimating the habitual frequency of consumption of high-fat foods.

The mean frequency of eating high-fat foods, as measured by the app (with or without the correction), did not differ significantly between normal weight and overweight/obese subjects (Table 2). The results from Q1 and the valid days from the application were correlated (r = 0.42, *p* < 0.001) but when we stratified the group by BMI, this was no longer significant among the overweight or obese people; in normal weight subjects, the correlation coefficient was = 0.67 (*p* < 0.001). The valid responses from the app and the Q2 results were positively correlated for the entire group and for the normal weight subjects (r = 0.38, *p* < 0.01 and r = 0.55, *p* < 0.01, respectively). There was no such correlation for the overweight or obese people. These correlations were only observed when the subset of valid responses was analyzed. Without the correction, the application data did not correlate with Q1 or Q2 in the entire group or in the body weight subgroups. Additionally, we stratified the group by sex. The valid responses from the app and the results from the Q1 and Q2 were positively correlated for men only (r = 0.47, *p* < 0.05 and r = 0.47, *p* < 0.05, respectively).

We further examined how the reported frequencies of high-fat food intake correlated with the total calorie intake and percentage energy from fat. The frequency of consumption of high-fat foods measured with Q1 and Q2 did not correlate with the total calorie intake and percentage energy from fat, either in the entire group or in the BMI subgroups. Interestingly, there was a correlation in the entire group between total energy intake and the frequency of consumption of high-fat foods as measured by the valid responses from the application (r = 0.55, *p* < 0.001). This mainly resulted from the strong positive correlation observed in overweight and obese people (r = 0.83, *p* < 0.001), as it was not found in people of normal weight. Moreover, a correlation between frequency of consumption of high-fat foods measured by the application (valid responses) and percentage energy from fat was observed only in overweight and obese people (r = 0.50, *p* < 0.05). There was no correlation between the entire data set from the application and percentage energy from fat.

**Figure 1:**
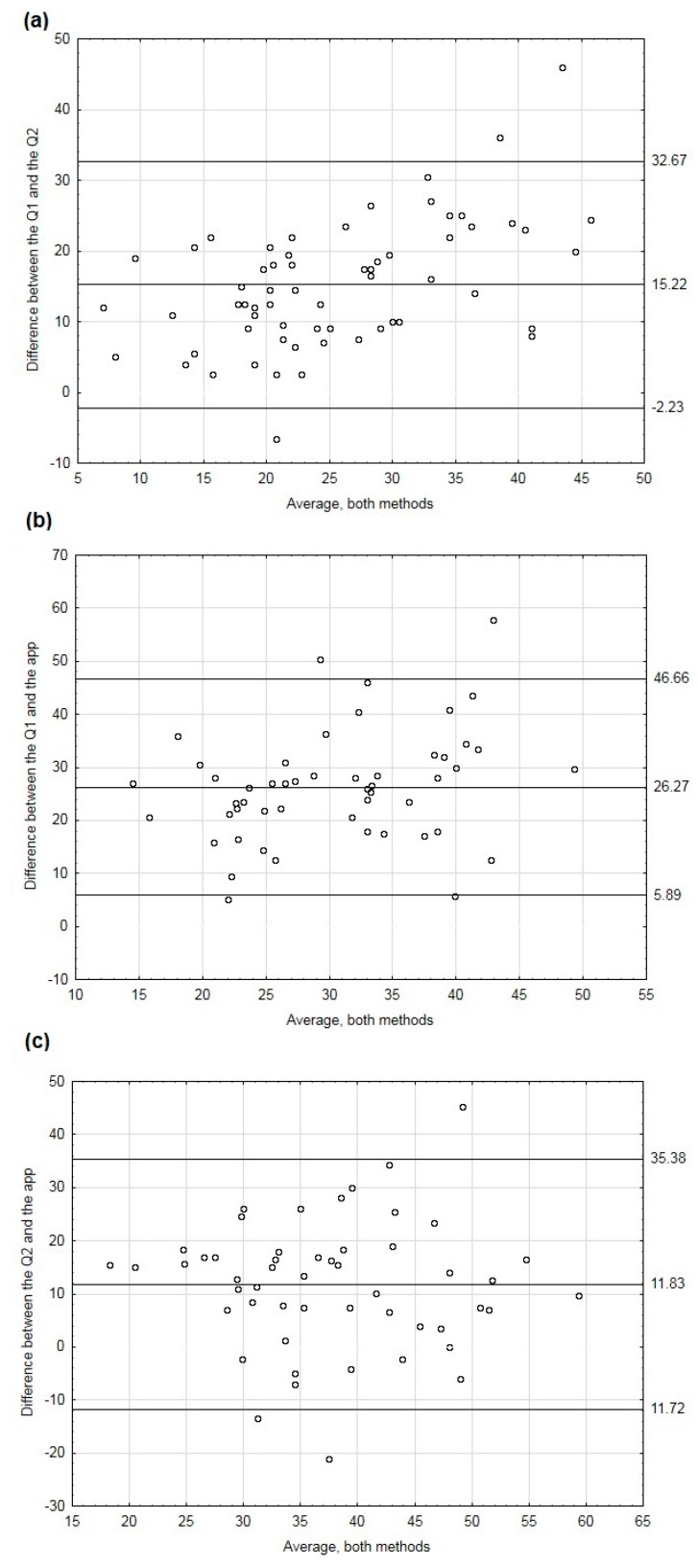
Bland–Altman plots of individual differences between Q1 and Q2 (**a**), Q1 and the app (**b**) and Q2 and the app (**c**). In case of the app, only data from valid responses were considered. Valid responses were those in which respondents gave replies to at least three prompts on at least five days.

**Figure 2:**
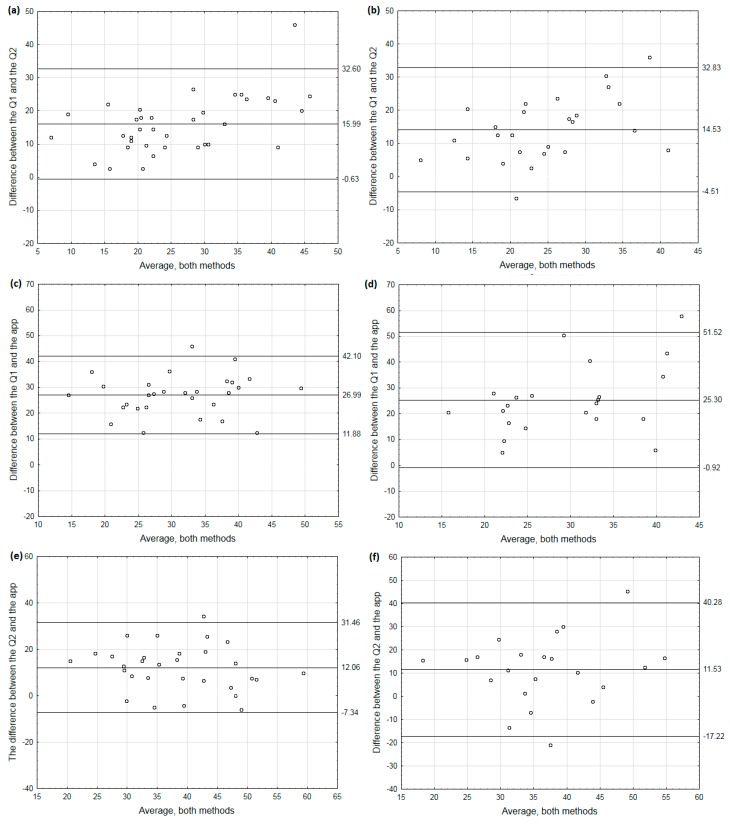
Bland–Altman plots of individual differences between Q1 and Q2, Q1 and the app and Q2 and the app in the normal weight (**a**,**c**,**e** respectively) and the overweight or obese individuals (**b**,**d**,**f**, respectively). In case of the app only data from valid responses were considered. Valid responses were those in which respondents gave replies to at least three prompts on at least five days.

Bland–Altman plots were used to illustrate the agreement between the methods. We first examined the difference between the entire data set from the app and the data set after correction. The mean agreement between Q1 and the app was 9.25 times/week (95% confidence interval: 33.29, −14.79) (Supplementary Figure S1). As data without correction seemed not to properly reflect eating behavior, we further analyzed the frequency of consumption of high-fat foods measured by Q1, Q2 and the app based only on the valid responses (Figure 1). The mean agreement between Q1 and the app was 26.27 times/week (95% confidence interval: 5.89, 46.66). We also performed Bland–Altman plots separately for people of normal weight and for those who were overweight and obese (Figure 2). The differences between Q1 and Q2 are very similar in both groups (Figure 2a,b). However, the differences between Q1 and the app were much higher in the overweight/obese group than in the normal weight group: the mean agreement being 25.30 (95% confidence interval: 51.52, −0.92) and 26.99 (95% confidence interval: 42.40, 11.88), respectively (Figure 2c,d). Similar results were obtained when we compared Q2 and the app (Figure 2e,f).

## 4. Discussion

This study evaluated an innovative method of measuring the frequency of consumption of high-fat foods by using an application for mobile devices and compared this application with a previously validated paper FFQ [7]. The study participants did not experience any problems using the app and the majority responded to the prompts at least once per day for the whole study period. However, to properly calculate the weekly frequency of consumption of high-fat foods and for further comparative analysis, we used only the data from subjects who gave replies to at least three prompts on at least five days. With this cut-off value, we captured 84% of the subjects, which is a satisfactory result, though lower than we had expected. A recent meta-analysis showed that a higher average compliance rate was observed in nonclinical studies that prompted participants 2–3 times daily than in those that prompted participants more frequently (4–5 times) [26]. The compliance rate for 4–5 prompts was around 77%, which agrees with our results. One of the reasons for the observed nonresponse in our study was that the app was tested in people aged 20–40, who had several activities during daytime during which they could not use phones, such as when they were working or studying. In this context, more than four prompts per day could lead to an even higher rate of nonresponse. The average prompting frequency in other EMA nonclinical studies was 4.2 [26] and this frequency seems the most reasonable and was used in our app. It should also be underlined that there was no difference in response rate between normal weight and overweight/obese subjects.

There are several commercial and research electronic applications. Many of them were designed to assess the total food consumption as they are electronic food diaries [21]. Although this approach seems promising, it nonetheless involves high burdens on participants [21,27] which depends on duration and frequency of prompts [26]. It could be thus assumed that the assessment of selected elements of dietary behavior (e.g., intake of a given group of food products) may enhance the data quality. As our primary goal was to increase the precision of data acquisition, we intended to focus on the frequency of consumption of high-fat foods only and we demonstrated here that the application is feasible and could be used to measure the frequency of consumption of this type of food in people aged 20–40. The EMA application can thus be used in a future study aimed at revealing the relationships between the frequency of consumption of high-fat foods and disease. In such a study, the intake of different types of high-fat foods should also be addressed, as it is well known that intake of foods rich in unsaturated fatty acids has different metabolic effects than intake of food product that are good sources of saturated fatty acids [28,29]. Our application is capable of measuring both these food types.

The results from the application were compared with data collected through paper FFQs, with shorter and longer lists of high-fat foods. We showed that the results from Q1 and Q2 were strongly positively correlated, while those from Q1 and the app were only moderately positively correlated. Additionally, when we analyzed people of normal weight and those who were overweight/obese separately, the positive correlation between Q1 and the app was only seen in the first group. As expected, the frequency of consumption of high-fat foods measured with Q2 was higher than that measured with Q1, with no difference between normal weight and overweight/obese subjects. The mean differences between Q1 and Q2 in the respective groups were 15.99 and 14.53 times/week. Although Q2 and the app used the same list of food items, the median frequency of consumption of high-fat foods measured with the app was about 40% higher than that measured with Q2. Moreover, the average difference between Q2 and the app was 11.83 times/week. Together, these results suggest that including additional food items in the FFQ does not affect the outcome significantly and reflects similar tendencies in normal weight people as well as in those who are overweight or obese. Additionally, as a retrospective method, the paper FFQ may lead to an underestimation of the real consumption frequency of high-fat foods as compared to an app measure. It has been shown that FFQs usually overestimate food intake [30] but the comparisons were made between two retrospective methods and it has never been tested how combining FFQ with EMA affects measurements.

Additionally, our results indicate that proper calculation of the frequency of consuming high-fat foods may require a cut-off method. The analysis included only days with a response to at least three prompts on at least five days. In this way, we excluded data from ten subjects, six of normal weight and four who were overweight/obese. There was no correlation between body weight status and the number of days on which people gave replies to at least three prompts. This suggests that nonresponse in our study might have been for random reasons. Additionally, data from the entire data set and the corrected data set were correlated (r = 0.9), which is consistent with other studies [31,32]. Interestingly, despite the correlation between these two data sets, correlations between the entire data set and percentage energy from fat were not observed. There are a few possible explanations for this. One of them is that simply the frequency of eating high-fat foods calculated from the entire dataset (valid and invalid responses) does not correspond well to reported percentage energy from fat. In this case invalid responses can be understood as outliers resulting from data collection errors and the presence of these outliers affects the correlations. Moreover, although missing data is a common problem in dietary assessment, it is frequently unreported how different studies have dealt with it [31]. In studies using FFQs, the common approach of assuming that nonresponse equates to not eating that particular food can introduce significant bias [31,33]. Using applications for dietary data collection gives the advantage of easy correction of data sets.

Several studies have shown that body weight status may influence reporting of food intake and dietary behaviors [34,35]. For this reason, we analyzed the results in subgroups stratified by BMI and the correlations between the frequency of consumption of high-fat foods and energy intake or percentage energy from fat measured from the dietary records differed between those groups. Interestingly, we found such correlations but only for the frequency of consumption of high-fat foods measured with the app. In overweight/obese individuals, there was a strong positive correlation between the frequency of consumption of high-fat foods and the total energy intake and also a moderate positive correlation with percentage energy from fat. Percentage energy from fat did not differ between groups. It is worth mentioning that, although Q2 contained the same list of food items as the app, the measures obtained with Q2 correlated neither with total energy intake nor with percentage energy from fat. The agreement between Q1 and Q2 was similar in normal weight people and in overweight and obese individuals. Moreover, the mean differences between Q2 and the app were similar in both groups but the agreement limits were much wider in the overweight/obese group than in the normal weight group (40.28, −17.22 vs. 31.46, −7.34). Interestingly, the bias between Q2 and the application seems to be independent of the range of intake in both groups. These results suggest that normal weight people estimated their frequency of consumption of high-fat foods well; in overweight/obese people, the use of an application that forces a response to a prompt in a real-time manner may be a promising approach for capturing high-fat food intake. Another explanation of the different results for the two body weight groups might be that one of the groups consumes high-fat foods more frequently, which may affect the accuracy of reporting. However, we have shown here that there was no difference in frequency of consumption of high-fat foods between normal weight and overweight/obese people.

Inaccurate data on food intake is a limitation of nutritional studies and may prevent an understanding of the impact of dietary factors on health and disease and inhibit the ability to assess the efficacy of dietary interventions [36]. This is also an oft-mentioned problem in association studies, where more accurate phenotyping strategies are required to fully characterize the contribution of genetic factors to phenotype variability [37]. Each of these methods of dietary intake assessment has its limitations, often leading to the underreporting of eating frequency concurrent with the underreporting of energy intake [38]. These limitations can be exaggerated in the context of gene × nutrient interactions in large-cohort studies [39]. The need for technological innovation in dietary assessment has long been postulated [40] and several approaches have been tested to date [39]. However, using applications for mobile devices in association studies is a new approach that should be introduced and validated.

Our study has several strengths. First, it is one of very few studies to use EMA in nutritional research, especially for intake assessment. Moreover, this is the first study to use an FFQ-based application in measuring the frequency of high-fat foods. The initial FFQ was a validated and commonly used questionnaire. A main limitation was that the study was conducted in a group of people who are capable of using mobile phones and apps but access to these technologies is not universal. Although the sample size was similar to that of earlier studies [27,41,42], it was relatively small. Additionally, we used several exclusion criteria and focused on healthy people, which also limits generalizability. Another possible limitation was that participants were unable to install the app on their own phones and for this reason they needed to carry two smartphones. Additionally, all FFQs should be validated for specific populations [4]. For this reason, our application would also require validation before it could be used in a different country.

## 5. Conclusions

Our study demonstrates that an application for mobile devices is a feasible tool for capturing the frequency of high-fat food consumption in both normal weight and overweight/obese people. However, the responses may be affected by body weight status. Comparison of the results from the paper questionnaire for assessing the consumption of high-fat foods and result from the EMA application showed that the retrospectively reported values were underestimated, compared with the measures made in real-life situations. Using a real-time assessment method seems to improve the measured frequency of consumption of high-fat foods, especially in overweight or obese people.

## Figures and Tables

**Table 1:** Characteristics of the study participants.

Characteristic	
	Mean ± SD
Age	25.8 ± 5.4
BMI [kg/m^2^]	25.41 ± 5.7
Gender	N (%)
Male	36 (58)
Female	26 (42)
Normal weight	26 (42)
Overweight or obese	36 (58)
Number of days when people responded on the app (at least once per day)	
10	11 (18)
9	43 (69)
8	4 (6)
7	1 (2)
6	2 (3)
5	0 (0)
4	1 (2)

BMI: body mass index; SD: standard deviation; N: sample size.

**Table 2:** Intake of high-fat foods measured with Q1, Q2 and the app and percentage energy from fat measured with dietary records. Data are presented as means with standard deviations.

Frequency of Consumption of High-Fat Foods Measured by Each Method	Group		
	Entire Group	Normal Weight Subjects	Overweight or Obese Subjects
	Mean ± SD	Mean ± SD	Mean ± SD
Q1 [times/week]	17.9 ± 8.0	18.2 ± 8.5 ^a^	17.4 ± 7.3 ^a^
Q2 [times/week]	33.1 ± 12.2	34.2 ± 2.8 ^a^	31.6 ± 1.3 ^a^
app [times/week]	49.6 ± 19.2	51.6 ± 21.5 ^a^	46.9 ± 15.5 ^a^
app corrected [times/week]	43.5 ± 10.7	44.5 ± 9.6 ^a^	42.2 ± 2.3 ^a^
% energy from fat	34.5 ± 8.0	35.6 ± 8.6 ^a^	33.0 ± 7.1 ^a^

^a^ Within the rows, the same superscript indicates no significant difference between the body weigh subgroups. Q1: questionnaire 1 which was Block Screening Questionnaire for Fat Intake; Q2: questionnaire 2; SD: standard deviation.

## Supplementary Materials

The following are available online at http://www.mdpi.com/2072-6643/10/11/1692/s1. Figure S1 Bland–Altman plot of individual differences between the app and the corrected app data. In the corrected app data, only valid days were considered. Valid responses were those in which respondents gave replies to at least three prompts on at least five days.
