# Supplementary figures and images for: Use of a Smartphone Application Can Improve Assessment of High-Fat Food Consumption in Overweight Individuals
Source: Nutrients. 2018 Nov 6;10(11):1692. doi: 10.3390/nu10111692 (PMC6266782; doi:10.3390/nu10111692)

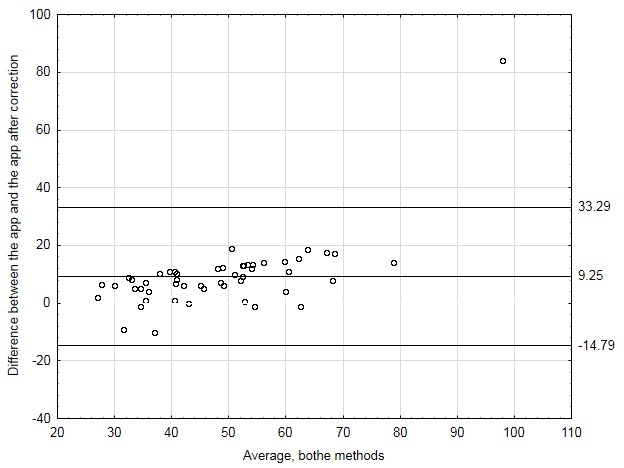

Supplement: Supplementary file 1 [file nutrients-10-01692-s001.jpg]
